# *Lactobacillus acidophilus* DDS-1 and *Bifidobacterium lactis* UABla-12 Improve Abdominal Pain Severity and Symptomology in Irritable Bowel Syndrome: Randomized Controlled Trial

**DOI:** 10.3390/nu12020363

**Published:** 2020-01-30

**Authors:** Christopher J. Martoni, Shalini Srivastava, Gregory J. Leyer

**Affiliations:** 1 UAS Laboratories LLC, 4375 Duraform Lane, Windsor, WI 53598, USA; cmartoni@uaslabs.com; 2 Vedic Lifesciences, 203 Morya Landmark1, New Link Road, Andheri W, Mumbai 400053, India; shalini.s@vediclifesciences.com

**Keywords:** Randomized controlled trial, irritable bowel syndrome, probiotic, abdominal pain, bowel habits, microbiome

## Abstract

This randomized, double-blind, placebo-controlled, multi-center study investigated the clinical efficacy of two probiotic strains on abdominal pain severity and symptomology in irritable bowel syndrome (IBS). Three hundred and thirty adults, aged 18 to 70 years, with IBS according to Rome IV criteria were allocated (1:1:1) to receive placebo, *Lactobacillus acidophilus* DDS-1 (1 × 10^10^ CFU/day) or *Bifidobacterium animalis* subsp. *lactis* UABla-12 (1 × 10^10^ CFU/day) over six weeks. The primary outcome was the change in Abdominal Pain Severity - Numeric Rating Scale (APS-NRS). Over the intervention period, APS-NRS was significantly improved in both probiotic groups vs. placebo in absolute terms (DDS-1: −2.59 ± 2.07, *p* = 0.001; UABla-12: −1.56 ± 1.83, *p* = 0.001) and in percentage of significant responders (DDS-1: 52.3%, *p* < 0.001); UABla-12 (28.2%, *p* = 0.031). Significant amelioration vs. placebo was observed in IBS Symptom Severity Scale (IBS-SSS) scores for *L. acidophilus* DDS-1 (−133.4 ± 95.19, *p* < 0.001) and *B. lactis* UABla-12 (−104.5 ± 96.08, *p* < 0.001) groups, including sub-scores related to abdominal pain, abdominal distension, bowel habits and quality of life. Additionally, a significant normalization was observed in stool consistency in both probiotic groups over time and as compared to placebo. In conclusion, *L. acidophilus* DDS-1 and *B. lactis* UABla-12 improved abdominal pain and symptom severity scores with a corresponding normalization of bowel habits in adults with IBS.

## 1. Introduction 

Irritable bowel syndrome (IBS), a functional disorder of the gastrointestinal tract, is characterized by chronic or recurrent abdominal pain and altered bowel habits [1]. Mechanisms include visceral hypersensitivity, altered bowel motility, abnormal epithelial barrier or intestinal permeability, immune activation, neurotransmitter imbalance, infection and dysbiosis of the microbiota [2,3]. Diagnosis of IBS is based on Rome IV criteria, a symptom-based classification system [4]. Current estimates report a worldwide prevalence of approximately 12%, making IBS the most common functional gastrointestinal disorder [4]. Clinical management is challenging due to the heterogeneity of symptoms, and no validated treatment algorithm exists [5].

Systematic reviews have demonstrated a limited but significant effect of probiotics over placebo on IBS symptoms [6,7,8,9]. Probiotics are defined as live microorganisms that, when administered in adequate amounts, confer a health benefit to the host [10]. A recent meta-analysis assessed 37 randomized controlled trials with a dichotomous outcome enrolling a total of 4403 participants [7,11]. Probiotics were deemed superior to placebo regarding global symptoms, albeit with a weak recommendation and low strength of evidence [11]. Significant heterogeneity between studies and frequent limitations in study quality, design and power have prevented an optimal probiotic strategy for individuals with IBS. As such, more well powered randomized controlled trials are warranted.

The present study is unique in its assessment of single-strain *Lactobacillus* (*acidophilus* DDS-1) and *Bifidobacterium* (*animalis* subsp. *lactis* UABla-12) probiotic arms simultaneously in a randomized controlled trial. *L. acidophilus* DDS-1, alone or in combination with *B. lactis* UABla-12, has previously been shown to normalize bowel habits in functional constipation [12], provide abdominal symptom relief in lactose intolerance [13] and support immune specific outcomes [14,15] in randomized controlled trials. Mechanistically, *L. acidophilus* DDS-1 has exhibited immunomodulatory capacity in vitro [16], while both modulating the microbiota and downregulating the inflammatory profile in pre-clinical models [17,18]. Additionally, *L. acidophilus* DDS-1 in combination with *B. lactis* UABla-12 has demonstrated an immune regulatory role in support of immune specific outcomes clinically [15]. Further, a probiotic blend including both *L. acidophilus* DDS-1 and *B. lactis* UABla-12 was previously shown in a pilot trial to improve symptomology in participants with IBS [19]. The above pre-clinical and clinical evidence provided a rationale for a well-powered randomized controlled trial in IBS, while individually assessing *L. acidophilus* DDS-1 and *B. lactis* UABla-12.

The current study focused on abdominal pain as the primary outcome as it is a defining characteristic of IBS and a driver of healthcare resource utilization [20]. Unlike most symptoms, such as altered bowel habits or bloating, abdominal pain has been shown to independently drive health-related quality of life decline in IBS and is the principal driver of patient-reported symptom severity [21,22].

Therefore, the current randomized, double-blind, placebo-controlled, parallel-arm, multi-center study was conducted to explore the efficacy and tolerability of *L. acidophilus* DDS-1 and *B. lactis* UABla-12, simultaneously, with respect to abdominal pain severity, IBS related symptomology and bowel habits in adults with IBS.

## 2. Subjects and Methods

### 2.1. Study Population

Healthy adults, aged 18 to 70 years, meeting Rome IV diagnostic criteria for IBS were recruited from outpatient settings of 12 clinics specializing in gastroenterology practice in Greater Mumbai, India. As per Rome IV, included participants had recurrent abdominal pain for the last 3 months with symptom onset at least 6 months before diagnosis, and at least two of the following three symptoms: pain related to defecation or change in stool frequency or stool form. Additionally, participants had an average maximal abdominal pain severity of ≥ 4 on the 11-point Abdominal Pain Severity -Numeric Rating Scale (APS-NRS) at screening and during the placebo run-in period as well as fasting blood glucose and hemoglobin levels at screening of ≤ 110 mg/dl and ≥ 10 g/dl, respectively. Participants with organic disease, a history of surgical resection of the stomach, small or large intestine, a history of inflammatory bowel disease, complications from infectious enteritis, hyperthyroidism or hypothyroidism, a history of diet-based intolerance, drug or alcohol abuse within the past 6 months, a history of malignant tumors, severe depression or anxiety disorder, or uncontrolled hypertension were excluded. Additional exclusion criteria included individuals with unstable medical conditions, type I or II diabetes, a history of cancer or abdominal surgery, immunocompromised conditions, or an active eating disorder. Smokers, defined as ≥ 1 cigarette a day and individuals consuming > 2 standard alcoholic drinks daily over the prior 3 months were excluded. The use of probiotic or fiber supplements, probiotic or fiber enriched foods, and IBS specific treatments were prohibited within 4 weeks of screening. Laxative medications or other agents affecting gastrointestinal motility were prohibited within 2 weeks of screening. All were prohibited during the trial. Lastly, individuals with an allergy or sensitivity to the study products and females who were pregnant, intended to get pregnant or breastfeeding were excluded.

### 2.2. Study Design

The study was conducted in accordance with the ethical principles that have their origins in the Declaration of Helsinki and its subsequent amendments. Study conduct was in full accordance with the study protocol and all applicable laws and regulations, including but not limited to current International Conference on Harmonisation - Good Clinical Practices, Schedule Y and the Indian Council of Medical Research Ethical Guidelines for Biomedical Research on Human Participants. The study was approved and monitored by an Independent Ethics Committee (Approval No: VED/P-01/03/FEB/2018; Address: ACEAS, Ahmedabad, Gujarat, India). The trial was registered on clinicaltrials.gov under study number NCT03482765 and was conducted according to the CONSORT 2010 Statement.

The study design was prospective, randomized, double-blind, placebo-controlled, multi-center and parallel-arm, including two active arms and one placebo arm. Participants visited the clinics at screening (day -18 ± 2), and if applicable, randomization (day 0), day 21 (± 2) and day 42 (± 2) of the six-week intervention period. At screening, the study design was explained to participants in their local language following which written, signed and dated informed consent was obtained. Participants were assessed for all inclusion/exclusion criteria and medical history, concomitant medication, demographic details and vitals were recorded. Participants meeting screening criteria were dispensed with placebo product for a 14-day run-in period.

At randomization, APS-NRS over the placebo run-in period was reconciled and the average was considered as the baseline score. A decrease of more than 25% in APS-NRS was classified as a placebo response and the participant was excluded from the study. Participants meeting the above criteria and exhibiting at least 80% of run-in product compliance were randomized. Participants were randomized at the ratio of 1:1:1 to receive either one of the investigational products (DDS-1 or UABla-12) or placebo based on the randomization schedule. Block randomization (block size: 6) was performed using Stats Direct software (version 3.2), generating distinct alphanumeric codes. Sites dispensed study products in sequential order of participant qualification. The randomization codes were concealed from investigators, as well as site and study teams involved in study conduct and evaluation, with blinding codes secured in tamper-evident sealed envelopes.

### 2.3. Study Product

The probiotic study products consisted of either *L. acidophilus* DDS^®^-1 or *B. animalis* subsp. *lactis* UABla-12™. Placebo and probiotic capsules (size “1” hypromellose) were prepared in compliance with standard operating and quality control procedures at UAS Laboratories LLC (Wausau, WI). *L. acidophilus* DDS-1 or *B. lactis* UABla-12 capsules, which each contained a potency of not less than (NLT) 1 × 10^10^ CFU/capsule, were formulated with lyophilized probiotic and microcrystalline cellulose. Placebo capsules were formulated with microcrystalline cellulose. All capsules contained minimal but identical quantities of magnesium stearate and silica as flow aids. Both finished formulations were non-allergenic. Probiotic and placebo capsules were identical in mass, taste and appearance and bottled in identical sealed 64 mL CSP bottles (CSP Technologies, Auburn, Alabama). Probiotic and placebo capsules were confirmed to meet all quality specifications, which included potency and bacterial culture purity, microbiological analyses, color, appearance, weight specification/variation and disintegration both at the time of manufacture and at the end of the clinical study period. Participants took the allotted study product orally, once daily before a meal, with a glass of water over the entire 42-day intervention period.

### 2.4. Outcome Assessments

Primary and secondary outcome assessments at baseline, midpoint and the end of study visits included abdominal pain severity, IBS symptom severity, stool form and frequency, IBS related quality of life and perceived stress. Additionally, a clinical examination was conducted, study product was dispensed, and study product compliance and concomitant medication usage was recorded. Mebeverine HCl (135 mg) as rescue medication to be taken in the case of severe pain was provided to the study participants for the length of the study.

The primary outcome was the change in abdominal pain severity over the intervention period. Abdominal pain severity was assessed via the 11-point APS-NRS [21], with 10 representing the most severe pain and 0 representing no pain. Post-screening APS-NRS scores were submitted using a digital diary on a weekly basis. An average of the prior 3 weeks’ score was considered as the score for the corresponding visit.

IBS symptom severity was assessed via the IBS Symptom Severity Scale (IBS-SSS) [23]. Scores on the IBS-SSS range from 0 to 500, with 5 individual domains (0 to 100) related to abdominal pain severity, frequency of abdominal pain, severity of abdominal distention, dissatisfaction with bowel habits, and interference with quality of life. A decrease of 95 points has been associated with a clinically meaningful improvement [21].

Stool consistency was assessed via the Bristol Stool Scale (BSS), a validated ordinal scale of stool types [24] ranging from 1 through 7, with types 1–2 and 6–7, in conjunction with other symptoms, indicative of constipation and diarrhea, respectively. Types 3–5 are generally considered to be the most normal stool form and are the modal stool forms in cross-sectional surveys of healthy adults.

IBS-related quality of life (QoL) was assessed via the IBS-QoL, a 34-item questionnaire with each item rated on a 5-point scale (range: 34–170), with increasing scores indicating deteriorating quality of life [25]. Mental stress was assessed using the Perceived Stress Scale (PSS), a validated psychological instrument measuring the perception of stress and comprising negative and positive domains [26].

### 2.5. Statistical Analysis

The sample size calculation was based on the primary outcome, abdominal pain severity. A reduction in APS-NRS of at least 15%, in comparison to placebo, was considered as a relevant treatment effect. Standard deviation (of the primary outcome) was estimated at 34%. A placebo effect was estimated at 20–25%. Assuming 80% study power and a two-sided statistical significance level of 5%, sample size was estimated to be approximately 82 participants per group (15% relative change compared to placebo). With an estimated drop-out rate of 15–20% after randomization, in this three-arm study, no less than 300 randomized participants were planned to be recruited at a ratio of 1:1:1, assuming a 15% relative change compared to placebo.

The primary and secondary objectives were assessed on the intention-to-treat (ITT) population. Descriptive statistics are presented as mean (standard deviation (SD)) or median (interquartile range (IQR)) for continuous variables or as a percentage for qualitative variables. Data normality was assessed using the Shapiro–Wilk test. Differences between groups for baseline characteristics were analyzed using a one-way ANOVA, a non-parametric Kruskal–Wallis test for continuous variables, or a Pearson Chi Square test for categorical variables. IBS-specific outcomes over the intervention period were determined to be sufficiently non-normally distributed. Therefore, differences between groups in IBS specific outcomes were assessed via a non-parametric Kruskal–Wallis test, followed by a Mann–Whitney U to assess differences between individual treatment arms. Between group comparisons for categorical values over the intervention period, including responder rate and stool consistency profile, were assessed via a Pearson Chi Square test. Intragroup change in stool type was assessed via a McNemar test. Data processing, tabulation of descriptive statistics, and calculation of inferential statistics were performed using Statistical Package for the Social Sciences (SPSS, IBM^®^) or Python 3.0 (Armonk, New York, USA).

## 3. Results

### 3.1. Study Parameters

**Figure 1:**
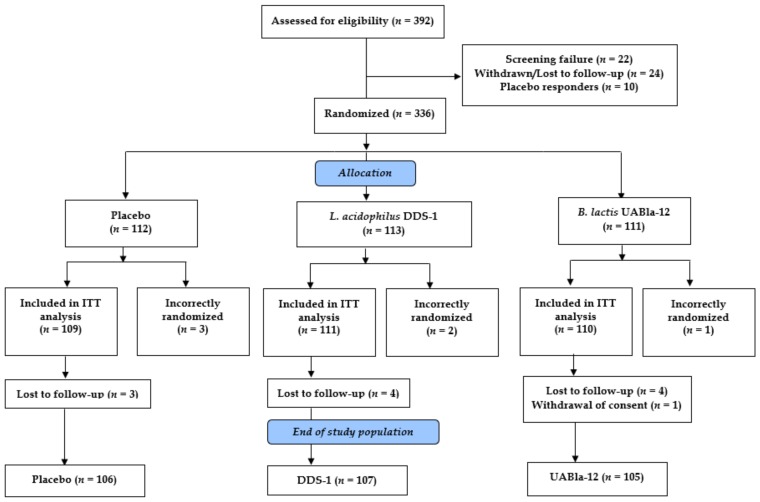
Participant flow chart. ITT: intention-to-treat.

A total of 392 potential participants were screened and 336 subjects were randomized in the study. Screening and enrollment occurred continuously from April 2018 through March 2019. Three hundred and thirty subjects were randomized as part of the ITT population at a ratio of 1:1:1 with 109 subjects in the placebo group, 111 subjects in the DDS-1 group and 110 subjects in the UABla-12 group (Figure 1). Six subjects were incorrectly enrolled without meeting entry criteria and were excluded. Eleven subjects were lost to follow-up and one subject withdrew from the study at their request. Three hundred and eighteen subjects completed the study per protocol, with 106, 107 and 105 subjects in the placebo, DDS-1 and UABla-12 groups, respectively.

### 3.2. Baseline Characteristics of Subjects

The baseline characteristics of the ITT population were evaluated and are presented in Table 1. The three groups were homogeneous in terms of demographic and clinical characteristics, including gender, body mass index, heart rate and blood pressure (*p* > 0.05). The mean age of subjects was higher in the *B. lactis* UABla-12 group (41.6 ± 11.1 years) as compared to the placebo group (37.6 ± 10.1 years). The ITT population comprised a nearly even distribution of females (46.6–52.3%) and males in all three groups. The participants were deemed to have IBS per Rome IV criteria, with a mean duration of 20.8 months, 20.9 months and 22.1 months in the placebo, *L. acidophilus* DDS-1 and *B. lactis* UABla-12 groups, respectively. Subjects in all three groups presented similar baseline scores (*p* > 0.05) related to anxiety (generalized anxiety questionnaire) and overall health (participant health questionnaire). No smokers were enrolled in the study. Additionally, alcohol consumption profiles were similar between groups (*p* > 0.05), with the vast majority of subjects not consuming alcohol. Dietary preferences were similar between groups with available evidence indicating a 2.1:1 ratio of non-vegetarians to vegetarians. All subjects enrolled in the trial were literate and living in urban areas.

### 3.3. Abdominal Pain Severity

**Figure 2:**
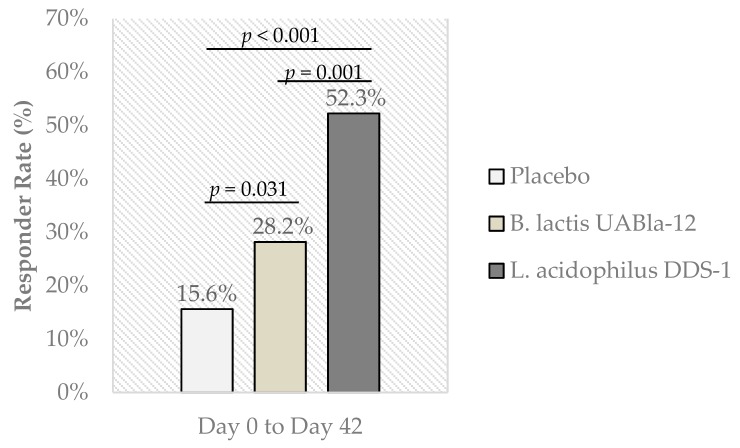
Percentage of significant responders, as defined by a decrease of 30% or more in the Abdominal Pain Severity – Numeric Rating Scale (APS-NRS) (primary outcome) from day 0 to day 42. Responder rate based on all subjects enrolled in ITT population. Between group comparison assessed by Pearson Chi Square test.

The groups were similar at baseline with mean APS-NRS scores of 6.94 ± 1.02, 7.03 ± 0.99 and 6.84 ± 1.04 for the placebo, *L. acidophilus* DDS-1 and *B. lactis* UABla-12 groups, respectively. Significant between-group differences were observed in the change in abdominal pain severity score (primary outcome) over the intervention period for both probiotic groups when compared to placebo (Table 2). At day 42, APS-NRS showed a greater reduction in *L. acidophilus* DDS-1 (−2.59 ± 2.07) and *B. lactis* UABla-12 (−1.56 ± 1.83) groups, as compared to placebo (−0.85 ± 1.45) (*p* = 0.001). *L. acidophilus* DDS-1 similarly showed a significant reduction at day 21 (−1.24 ± 1.10) as compared to placebo (−0.71 ± 0.92) (*p* < 0.001). Individuals considered to be significant responders to intervention, as defined by a greater than 30% reduction in abdominal pain severity, were significantly more numerous in *L. acidophilus* DDS-1 (52.3%, *p* < 0.001) and *B. lactis* UABla-12 (28.2%, *p* = 0.031) groups as compared to placebo (15.6%) (Figure 2).

### 3.4. IBS Symptomology

IBS-SSS scores were similar at baseline with mean scores of 298.07 ± 55.68, 310.90 ± 52.47 and 305.45 ± 48.82 for placebo, *L. acidophilus* DDS-1 and *B. lactis* UABla-12 groups, respectively (Table 3). Over the intervention period, subjects receiving *L. acidophilus* DDS-1 capsules reported significant reductions as compared to placebo in IBS-SSS (−133.4 ± 95.19, *p* < 0.001) with domain-specific scores related to abdominal pain severity (−32.94 ± 21.78, *p* < 0.001), abdominal pain duration (−28.44 ± 24.80, *p* < 0.001), abdominal distension (−27.20 ± 25.15, *p* < 0.001), bowel habits (−22.89 ± 22.46, *p* < 0.001) and quality of life (−21.88 ± 22.27, *p* < 0.001). Significant differences between *L. acidophilus* DDS-1 and placebo were also observed after 21 days for IBS-SSS total score (−56.06 ± 57.64, *p* < 0.001) and domains related to abdominal pain severity (−15.50 ± 15.23, *p* = 0.001), abdominal pain duration (−13.49 ± 21.79, *p* = 0.002), bowel habits (−8.90 ± 13.82, *p* = 0.007) and quality of life (−6.70 ± 14.58, *p* = 0.043). Over the intervention period, subjects taking *B. lactis* UABla-12 reported significant reductions as compared to placebo in IBS-SSS (−104.5 ± 96.08, *p* < 0.001) with domain-specific scores related to abdominal pain severity (−24.52 ± 24.35, *p* < 0.001), abdominal pain duration (-20.95 ± 23.91, *p* < 0.001), abdominal distension (−21.90 ± 25.85, *p* = 0.034) bowel habits (−17.86 ± 22.06, *p* = 0.001) and quality of life (−19.29 ± 21.66, *p* = 0.001). The *B. lactis* UABla-12 group also reported significant changes compared to placebo at the midpoint visit in IBS-SSS total score and domains related to abdominal pain severity, abdominal pain duration and bowel habits (*p* < 0.05).

### 3.5. Bowel Habits

Participants in both probiotic groups showed a significant normalization in stool consistency over the intervention period when compared to placebo. As shown in Table 4, all three groups exhibited similar distributions in stool type at baseline. A significantly different distribution was observed in *L. acidophilus* DDS-1 (*p* = 0.002) and *B. lactis* UABla-12 (*p* = 0.022) groups at day 42 as compared to placebo, with a higher percentage of subjects exhibiting normal stool type (83.8% or 75.5%, respectively) and a correspondingly lower percentage of subjects with constipation and diarrhea stool types (Table 4).

Normalization of stool type was also observed via intragroup changes, as shown in Table 5, which tracked individual movements of stool types over the intervention period. The placebo group exhibited a roughly equal number of subjects transitioning from non-normal to normal vs. normal to non-normal stool types over the intervention period (*p* = 1.000). In contrast, the *L. acidophilus* DDS-1 group showed a noticeable increase in participants transitioning to normal stool types at both day 21 (*p* = 0.006) and day 42 (*p* < 0.001), while the *B. lactis* UABla-12 showed a noticeable increase in participants transitioning to normal stool types at day 42 (*p* = 0.002).

A slight decrease in the daily number of stools was observed in both probiotic groups over the intervention period, however, no significant between-group differences were observed in stool frequency at any of the time points (Table S1).

### 3.6. Quality of Life, Perceived Stress and Product Tolerability

IBS-QoL scores were significantly improved in participants taking *L. acidophilus* DDS-1 as compared to placebo at the end of study visit (*p* = 0.016) (Table S2). PSS total scores were similarly improved at both day 21 (*p* = 0.002) and day 42 (*p* = 0.023) visits in subjects taking *L. acidophilus* DDS-1 as compared to placebo. Effects appeared to be more pronounced in sub-scores related to positive factors as compared to negative factors. In participants taking *B. lactis* UABla-12, despite intragroup improvement in IBS-QoL, no significant differences were observed when compared to placebo. Additionally, a significant effect was observed between *B. lactis* UABla-12 and placebo in PSS at day 21 (*p* = 0.030), however, no difference was observed at the end of study visit.

No differences were observed in study product tolerability between placebo and probiotic groups over the intervention period (Table S3). Similarly, no differences were observed in study product compliance, with mean compliance rates over the 42 day intervention period of 99%, 100% and 100% for the placebo, *L. acidophilus* DDS-1 and *B. lactis* UABla-12 groups, respectively (Table S3). Use of rescue medication was comparable and relatively rare, with approximately 90% of subjects not recording any use over the study period in all three groups (*p* > 0.05). Additionally, no significant changes were observed in safety variables in any group over the study period (Table S4).

## 4. Discussion

The present study was a randomized, double-blind, placebo-controlled trial to assess two probiotic strains, *L. acidophilus* DDS-1 and *B. animalis* subsp. *lactis* UABla-12, in adults with IBS. The study was unique in its simultaneous and independent assessment of a single strain *Lactobacillus* and *Bifidobacterium* in a well-powered three-arm trial of IBS patients across 12 clinical sites. A dose of NLT 1 × 10^10^ CFU daily was administered as it is supported by prior gastrointestinal focused randomized controlled trials on the strains [12,13] and is a midpoint dosage in meta-analyses of probiotic studies for IBS [9]. Enrolled participants were primarily young to middle-aged adults, with a mixed gender allocation and a normal to overweight BMI, representing a typical cross-section of IBS in India and elsewhere [27]. The lack of an obvious female predominance among IBS subjects in this study is in agreement with other Asian reports, and in contrast to a 2–3-fold greater prevalence among females in Western populations [27,28]. There is an unmet need for alleviation of IBS symptoms in Indian clinical practices, particularly in urban areas. Chronic gut infections, gut microbiota dysbiosis, and small intestinal bacterial overgrowth are suggested as contributing factors in South Asian populations [28].

Both strains achieved the primary outcome, reducing abdominal pain severity over the intervention period, when compared to placebo. The US Food and Drug Administration (FDA) has recommended the use of the 11-point numeric rating scale for abdominal pain severity, assessed here as primary outcome, for pain in IBS [29]. Further, the APS-NRS is both well validated and can be readily interpreted with a clinically important difference in IBS [21].

An intervention period of six weeks was assessed for the primary outcome, as it is consistent with the four- to eight-week probiotic IBS study durations widely reported in the literature [9]. Placebo response is significant in IBS studies and has been demonstrated to be highest in trials with a one- to four-week intervention period [30]. An initial increase in placebo response has been reported before stabilizing over two to five weeks and decreasing after 12 weeks [31,32]. For this reason, it has been suggested to include an intervention period of at least five weeks, and preferably longer, to avoid a potential unstable placebo group response [33]. Alternatively, other reviews report a higher placebo response associated with longer treatment duration and a greater number of study visits [34]. While the current intervention period was relatively short at six weeks, it incorporated design considerations [33], including a placebo run-in period, exclusion criteria regarding placebo responders, relatively few study visits and a large sample size to ensure adequate power.

FDA guidance defines a responder as a decrease in weekly average abdominal pain of at least 30% compared to baseline [29]. Clinically significant responders in the current study, considering the entire ITT population including dropouts, were 52.3% and 28.2% in the *L. acidophilus* DDS-1 and *B. lactis* UABla-12 groups respectively, both of which were significantly higher than placebo (15.6%). It is of interest that both probiotic strains, most notably *L. acidophilus* DDS-1, showed a significant group response as well an individual response to intervention. The meaningfulness of individual responses to treatment may, in some cases, be of more interest than group responses [35], and thus both are reported here. The high responder rates observed herein may also be applicable more generally. Episodes of abdominal pain occur in healthy people as well as individuals with IBS with the difference being the frequency and severity of symptoms [36]. Therefore, according to the European Food Safety Authority, IBS patients are generally an appropriate study group to substantiate claims related to gastrointestinal discomfort in the general population [36].

Participants taking *L. acidophilus* DDS-1 and *B. lactis* UABla-12 exhibited normalization of stool type in concert with improved outcomes in abdominal pain severity. Previously, a probiotic blend including the two strains studied here helped modulate bowel habits in functional constipation [12]. In the current study, a reduction in both constipation and diarrhea related stool types and a corresponding increase in normal stool types was observed, most noticeably with *L. acidophilus* DDS-1. A significant change in stool type was observed despite no significant alteration in stool frequency. Basal levels of stool frequency tend to be relatively higher in India [27]. Further, a patient’s description of constipation or diarrhea based on stool frequency alone can often be erroneous [37]. It has therefore been suggested to include stool type for a better assessment of bowel patterns in Indian populations [37].

Both probiotic strains helped improve total symptomology as per the validated questionnaire, with changes that could be classified as clinically significant. It has been reported that a minimally important clinical difference for the IBS-SSS is 95 points and thus patients exceeding this change could be classified as a responder [21]. Domains related to abdominal pain severity and duration, bloating, bowel habits and quality of life were also improved in probiotic groups as compared to placebo.

Multiple reports have linked IBS pathogenesis to dysbiosis of the microbiota [38,39], including low levels of *Lactobacillus* and *Bifidobacterium* [40], which could otherwise help inhibit binding of pathogens and reinforce defenses of the mucosal barrier. The Rome Team Working Group has supported the concept that the intestinal microbiota is perturbed in IBS [41]. There is evidence of an activation of the intestinal immune system in IBS resulting in inflammation, including augmented mucosal intraepithelial lymphocytes and mast cells [42,43].

The gut microbiota also plays an important role in the balance between immunosuppression and inflammation, involving Toll-like receptor signaling pathways [39]. *L. acidophilus* DDS-1 was previously shown to help modulate the fecal and mucosal microbiota in young and aging mice, while downregulating the production of inflammatory cytokines in serum and colonic explants [17,18]. Additionally, *L. acidophilus* DDS-1, in combination with *B. lactis* UABla-12, displayed improved symptomology associated with atopic dermatitis in a randomized controlled trial, with modulation of blood lymphocyte subsets (i.e., CD4, CD8 and CD25) suggesting an immune regulatory role [15].

In addition to the above, the microbiota can modulate visceral afferent pathways by effecting enterocytes, enteroendocrine cells, and the neurons themselves [44]. *L. acidophilus* has been shown to normalize visceral pain responses via induced expression of opioid and cannabinoid receptors in the gut [45]. Similarly, the species has been shown to modulate µ-opioid receptor expression and activity, which has been suggested as a mechanism for the reduction of bloating in adults with functional abdominal pain [46,47]. More recently, *Lactobacillus* was shown to upregulate expression of both serotonin transporter and intestinal serotonin levels [48], which further suggests a potential gut-brain role. In addition to the regulation of pain, mental stress was decreased with *L. acidophilus* DDS-1 in the current study. As IBS is considered a stress sensitive disorder [49], improved stress levels are possibly linked to a more normalized immune response, intestinal motility or barrier function.

Limitations of this study include the absence of microbial profiling or biomarker analysis, in part due to the large enrollment across many clinical sites. Further, while dietary guidelines were provided to subjects throughout the study, a dietary analysis was not performed. A recent study demonstrated a unique composition of the Indian gut microbiome, including diverging profiles of cohorts from North-Central and South India, who were primarily consuming plant-based and omnivorous diets, respectively [50]. Based on available evidence for the current study, omnivorous diets outnumbered plant-based diets in all three groups, with an overall ratio of 2.1 to 1. Correlation of the microbial community with diet and other metadata in the context of responders and non-responders would have been of interest to explore given the results of the current study.

Nevertheless, the study was well-powered to assess its primary and secondary outcome, it took into account integrated study design considerations for IBS [33], including a placebo run-in period and alignment of the study inclusion with the study outcomes, and outcomes were assessed over numerous clinical sites. In conclusion, abdominal pain severity was significantly improved in subjects receiving *L. acidophilus* DDS-1 or *B. lactis* UABla-12, both in terms of group response and individual response to intervention. Further, both probiotic strains showed a clinically significant alleviation of symptom severity with a corresponding normalization of bowel habits in males and females with IBS.

## Figures and Tables

**Table 1:** Baseline demographics and clinical characteristics of the ITT population.

	Placebo (*n* = 109)	*L. acidophilus* DDS-1 (*n* = 111)	*B. lactis* UABla-12 (*n* = 110)	*p* Value
	Mean (SD) or *n* (%)	Mean (SD) or *n* (%)	Mean (SD) or *n* (%)	
Age (years)	37.61 (10.12)	39.41 (11.80)	41.60 (11.11)	0.028 ^‡^
Female gender, *n* (%)	54 (49.5)	58 (52.3)	51 (46.6)	0.681 ^†^
BMI (kg/m^2^)	24.10 (4.06)	24.09 (4.34)	23.78 (3.98)	0.811 ^‡^
Systolic BP (kPa)	15.89 (1.13)	15.96 (1.08)	16.03 (1.11)	0.660 ^§^
Diastolic BP (kPa)	10.09 (0.94)	10.33 (0.86)	10.25 (1.00)	0.116 ^§^
Pulse Rate (bpm)	78.23 (8.63)	76.96 (7.69)	78.18 (8.10)	0.426 ^‡^
IBS duration (months)	20.81 (10.92)	20.85 (12.29)	22.07 (12.80)	0.674 ^‡^
GAD score	3.88 (1.81)	4.04 (1.62)	4.16 (1.64)	0.378 ^§^
PHQ-9 score	5.38 (3.15)	5.61 (3.05)	5.32 (2.96)	0.749 ^‡^
Alcohol consumption				
None, *n* (%)	96 (88.1%)	100 (90.1%)	97 (88.2%)	
Occasional, *n* (%)	3 (2.8%)	2 (1.8%)	3 (2.7%)	0.983 ^†^
Not available, *n* (%)	10 (9.2%)	9 (8.1%)	10 (9.1%)	

^†^ Between group comparison, Pearson Chi Square test; ^‡^ Between group comparison, one-way ANOVA; ^§^ Between group comparison, Kruskal–Wallis test; BMI: body mass index; BP: blood pressure; IBS: irritable bowel syndrome; GAD: generalized anxiety disorder; PHQ: participant health questionnaire; SD: standard deviation.

**Table 2:** Abdominal pain severity scores over the intervention period.

	Mean (SD)			*p* Value ^†^		
	Placebo (*n* = 109)	*L. acidophilus* DDS-1 (*n* = 111)	*B. lactis* UABla-12 (*n* = 110)	DDS-1 vs. Placebo	UABla-12 vs. Placebo	DDS-1 vs. UABla-12
APS-NRS Score						
Day 0	6.94 (1.02)	7.03 (0.99)	6.84 (1.04)	0.499	0.412	0.155
AbsΔ (Day 21)	−0.71 (0.92)	−1.24 (1.10)	−0.88 (0.99)	< 0.001	0.178	0.012
AbsΔ (Day 42)	−0.85 (1.45)	−2.59 (2.07)	−1.56 (1.83)	0.001	0.001	< 0.001

^†^ Between group comparison, Mann–Whitney U test; APS-NRS: Abdominal Pain Severity – Numeric Rating Scale; AbsΔ: absolute change.

**Table 3:** IBS symptom severity total and domain specific scores over the intervention period.

	Mean (SD)			*p* Value ^†^		
	Placebo (*n* = 109)	*L. acidophilus* DDS-1 (*n* = 111)	*B. lactis* UABla-12 (*n* = 110)	DDS-1 vs. Placebo	UABla-12 vs. Placebo	DDS-1 vs. UABla-12
IBS-SSS Total Score						
Day 0	298.07 (55.68)	310.90 (52.47)	305.45 (48.82)	0.108	0.324	0.504
AbsΔ (Day 21)	−30.09 (57.76)	−56.06 (57.64)	−50.00 (63.72)	< 0.001	0.020	0.185
AbsΔ (Day 42)	−55.70 (86.42)	−133.4 (95.19)	−104.5 (96.08)	< 0.001	< 0.001	0.039
Abdominal Pain Severity						
Day 0	66.74 (11.39)	68.24 (11.73)	65.82 (11.30)	0.213	0.644	0.075
AbsΔ (Day 21)	−9.25 (15.09)	−15.50 (15.23)	−12.86 (16.36)	0.001	0.077	0.121
AbsΔ (Day 42)	−13.41 (20.17)	−32.94 (21.78)	−24.52 (24.35)	< 0.001	< 0.001	0.009
Abdominal Pain Duration						
Day 0	53.21 (15.69)	59.73 (18.85)	57.55 (17.57)	0.016	0.139	0.344
AbsΔ (Day 21)	−4.77 (17.72)	−13.49 (21.79)	−10.95 (19.83)	0.002	0.023	0.420
AbsΔ (Day 42)	−8.13 (19.53)	−28.44 (24.80)	−20.95 (23.19)	< 0.001	< 0.001	0.018
Abdominal Distension						
Day 0	58.39 (17.81)	60.14 (16.96)	58.23 (17.79)	0.428	0.822	0.247
AbsΔ (Day 21)	−7.66 (20.39)	−11.47 (16.26)	−9.67 (18.92)	0.110	0.433	0.396
AbsΔ (Day 42)	−15.47 (23.81)	−27.20 (25.15)	−21.90 (25.85)	< 0.001	0.034	0.098
Bowel Habits						
Day 0	60.05 (14.38)	63.06 (13.79)	63.09 (12.06)	0.122	0.119	0.974
AbsΔ (Day 21)	−4.11 (13.95)	−8.90 (13.82)	−8.86 (14.99)	0.007	0.010	0.999
AbsΔ (Day 42)	−7.99 (23.46)	−22.89 (22.46)	−17.86 (22.06)	< 0.001	0.001	0.067
Effect on Quality of Life						
Day 0	59.68 (14.57)	59.73 (13.70)	60.77 (11.62)	0.985	0.657	0.496
AbsΔ (Day 21)	−4.30 (15.76)	−6.70 (14.58)	−7.67 (15.61)	0.043	0.112	0.663
AbsΔ (Day 42)	−10.70 (21.98)	−21.88 (22.27)	−19.29 (21.66)	< 0.001	0.001	0.316

^†^ Between group comparison, Mann–Whitney U test; IBS-SSS: Irritable Bowel Syndrome – Severity Scoring System.

**Table 4:** Stool consistency profile over the intervention period.

		Subjects (*n*, %)		
Group	Stool type (BSS)	Day 0	Day 21	Day 42
Placebo (*n* = 109)	Constipation (1–2)	25 (22.9)	19 (17.4)	28 (25.7)
	Normal (3–5)	70 (64.2)	74 (67.9)	68 (62.4)
	Diarrhea (6–7)	14 (12.8)	14 (12.8)	11 (10.1)
	Data not available	0	2 (1.8)	2 (1.8)
*L. acidophilus* DDS-1 (*n* = 111)	Constipation (1–2)	24 (21.6)	14 (12.6)	9 (8.1)
	Normal (3–5)	63 (56.8)	82 (73.9)	93 (83.8)
	Diarrhea (6–7)	24 (21.6)	13 (11.7)	7 (6.3)
	Data not available	0	2 (1.8)	2 (1.8)
*B. lactis* UABla-12 (*n* = 110)	Constipation (1–2)	22 (20.0)	18 (16.4)	10 (9.1)
	Normal (3–5)	67 (60.9)	63 (57.3)	83 (75.5)
	Diarrhea (6–7)	21 (19.1)	24 (21.8)	12 (10.9)
	Data not available	0	5 (4.5)	5 (4.5)
*p* Value ^†^	DDS-1 vs. Placebo	0.234	0.733	0.002
	UABla-12 vs. Placebo	0.438	0.444	0.022
	DDS-1 vs. UABla-12	0.139	0.234	0.435

^†^ Between group comparison, Pearson Chi Square test. BSS: Bristol Stool Scale.

**Table 5:** Intragroup change in stool type over the intervention period.

		Subjects (*n*, %)		*p* (Within Group) ^†^	
Group	Change in Stool Type	Day 0 to 21	Day 0 to 42	Day 0 to 21	Day 0 to 42
Placebo (*n* = 107)	Non-normal to Normal	21 (19.6)	21 (19.6)	0.511	1.000
	Non-normal to Non-normal	17 (15.9)	17 (15.9)		
	Normal to Non-Normal	16 (15.0)	22 (20.6)		
	Normal to Normal	53 (49.5)	47 (43.9)		
*L. acidophilus* DDS-1 (*n* = 109)	Non-normal to Normal	31 (28.4)	38 (34.9)	0.006	< 0.001
	Non-normal to Non-normal	15 (13.8)	8 (7.3)		
	Normal to Non-Normal	12 (11.0)	8 (7.3)		
	Normal to Normal	51 (46.8)	55 (50.5)		
*B. lactis* UABla-12 (*n* = 105)	Non-normal to Normal	16 (15.2)	28 (26.7)	1.000	0.002
	Non-normal to Non-normal	26 (24.8)	14 (13.3)		
	Normal to Non-Normal	16 (15.2)	8 (7.6)		
	Normal to Normal	47 (44.8)	55 (52.4)		

^†^ Within group comparison, McNemar test; Normal stool type (3–5). Non-normal stool type (1,2 or 6,7).

## Supplementary Materials

The following are available online at https://www.mdpi.com/2072-6643/12/2/363/s1, Table S1: Daily stool frequency over intervention period Table S2: IBS quality of life and perceived stress scores over intervention period; Table S3: Study product tolerability and compliance over intervention period; Table S4: Safety variables over intervention period.
