# Supplementary material for: Lactobacillus acidophilus DDS-1 and Bifidobacterium lactis UABla-12 Improve Abdominal Pain Severity and Symptomology in Irritable Bowel Syndrome: Randomized Controlled Trial
Source: Nutrients. 2020 Jan 30;12(2):363. doi: 10.3390/nu12020363 (PMC7071206; doi:10.3390/nu12020363)
Supplement: Supplementary file 1 [file nutrients-12-00363-s001.pdf]

Table S1. Daily stool frequency over intervention period

		Placebo (n = 109)	*L. acidophilus* DDS-1 (n = 111)	*B. lactis* UABla-12 (n = 110)	p Value †
Stool Frequency					
Day 0	Mean (SD)	2.7 (1.4)	2.7 (1.5)	2.7 (1.4)	0.530
	Median (Range)	2.2 (0.4-6.0)	2.1 (0.4-6.1)	2.3 (0.5-6.2)	
Day 21	Mean (SD)	2.7 (1.5)	2.6 (1.5)	2.6 (1.4)	0.187
	Median (Range)	2.3 (0.6-6.1)	2.0 (0.4-6.0)	2.2 (0.4-6.2)	
Day 42	Mean (SD)	2.8 (1.5)	2.3 (1.4)	2.4 (1.2)	0.410
	Median (Range)	2.3 (0.4-5.6)	1.7 (0.4-5.8)	2.2 (0.4-5.5)	

†Between group comparison, Pearson Chi Square U test

Table S2. IBS quality of life and perceived stress scores over intervention period

	Mean (SD)			*p*Value†		
	Placebo (*n* = 109)	*L. acidophilus* DDS-1 (*n* = 111)	*B. lactis* UABla-12 (*n* = 110)	DDS-1 vs. Placebo	UABla-12 vs. Placebo	DDS-1 vs. UABla-12
IBS-QoL Total Score						
Day 0	66.17 (18.26)	68.21 (22.35)	68.12 (21.52)	0.882	0.786	0.939
Day 21	62.21 (17.80)	62.51 (20.97)	63.88 (19.76)	0.762	0.723	0.462
Day 42	59.68 (19.40)	53.94 (17.97)	58.45 (21.91)	0.016	0.367	0.130
PSS Total Score						
Day 0	16.07 (6.42)	15.92 (7.12)	15.71 (6.77)	0.253	0.691	0.585
Day 21	16.04 (6.49)	13.89 (6.68)	14.80 (7.06)	0.002	0.030	0.356
Day 42	14.67 (6.61)	13.06 (6.85)	13.79 (7.13)	0.023	0.238	0.306
PSS Negative Factors						
Day 0	9.30 (4.62)	9.53 (4.83)	9.16 (4.40)	0.745	0.756	0.563
Day 21	9.05 (4.48)	8.15 (4.24)	8.77 (4.61)	0.118	0.578	0.336
Day 42	8.43 (4.38)	7.67 (4.30)	8.00 (4.53)	0.160	0.342	0.518
PSS Positive Factors						
Day 0	6.77 (2.75)	6.39 (2.94)	6.55 (2.98)	0.772	0.741	0.940
Day 21	6.99 (2.96)	5.74 (2.80)	6.03 (2.97)	0.014	0.192	0.275
Day 42	6.24 (2.99)	5.39 (3.12)	5.79 (3.10)	0.051	0.186	0.427

†Between group comparison, Mann Whitney U test

IBS-QoL: Irritable Bowel Syndrome-Quality of Life; PSS: Perceived Stress Scale;

Table S3. Study product tolerability and compliance over intervention period

		Placebo	*L. acidophilus* DDS-1	*B. lactis* UABla-12	*p*Value
	Good	76 (71.7)	73 (68.2)	71 (67.6)	
Tolerability, *n* (%)	Average	28 (26.4)	30 (28.0)	30 (28.6)	0.753†
	Poor	2 (1.9)	4 (3.7)	4 (3.8)	
Compliance, Mean (SD)		0.99 (0.02)	1.00 (0.01)	1.00 (0.01)	0.846‡

†Between group comparison, Pearson Chi Square test

‡Between group comparison, One Way Anova

Table S4. Safety variables over intervention period

	Placebo (*n* = 109)	*L. acidophilus* DDS-1 (*n* = 111)	*B. lactis* UABla-12 (*n* = 110)
	Mean (SD)	Mean (SD)	Mean (SD)
Systolic Blood Pressure			
Day 0	119.42 (8.28)	119.80 (9.03)	119.87 (8.31)
Day 21	119.86 (7.71)	119.54 (8.06)	121.09 (7.75)
Day 42	118.75 (7.56)	119.10 (7.43)	119.87 (7.44)
p (Day 0 – 21)†	0.479	0.723	0.066
p (Day 0 – 42)†	0.356	0.288	1.000
Diastolic Blood Pressure			
Day 0	75.81 (7.07)	77.43 (6.49)	76.73 (7.15)
Day 21	75.80 (6.98)	76.84 (6.89)	76.80 (7.16)
Day 42	75.77 (6.90)	77.22 (5.83)	76.49 (6.63)
p (Day 0 – 21)†	0.989	0.320	0.909
p (Day 0 – 42)†	0.939	0.738	0.678
Pulse Rate			
Day 0	78.15 (8.70)	76.96 (7.73)	78.26 (8.16)
Day 21	77.01 (8.09)	77.64 (7.13)	78.17 (7.89)
Day 42	77.36 (7.10)	77.42 (7.70)	78.89 (7.99)
p (Day 0 – 21)†	0.076	0.183	0.878
p (Day 0 – 42)†	0.242	0.444	0.348

†Within group comparison, Paired t-test
